# Characteristic distribution of finite-time Lyapunov exponents for chimera states

**DOI:** 10.1038/srep29213

**Published:** 2016-07-04

**Authors:** André E. Botha

**Affiliations:** 1 Department of Physics, University of South Africa, Science Campus, Private Bag X6, Florida 1710, South Africa

## Abstract

Our fascination with chimera states stems partially from the somewhat paradoxical, yet fundamental trait of identical, and identically coupled, oscillators to split into spatially separated, coherently and incoherently oscillating groups. While the list of systems for which various types of chimeras have already been detected continues to grow, there is a corresponding increase in the number of mathematical analyses aimed at elucidating the fundamental reasons for this surprising behaviour. Based on the model systems, there are strong indications that chimera states may generally be ubiquitous in naturally occurring systems containing large numbers of coupled oscillators – certain biological systems and high-*T*_*c*_ superconducting materials, for example. In this work we suggest a new way of detecting and characterising chimera states. Specifically, it is shown that the probability densities of finite-time Lyapunov exponents, corresponding to chimera states, have a definite characteristic shape. Such distributions could be used as signatures of chimera states, particularly in systems for which the phases of all the oscillators cannot be measured directly. For such cases, we suggest that chimera states could perhaps be detected by reconstructing the characteristic distribution via standard embedding techniques, thus making it possible to detect chimera states in systems where they could otherwise exist unnoticed.

Lyapunov characteristic exponents^1^,^2^, or more briefly Lyapunov exponents (LEs)^3^ characterise the time-averaged exponential divergence (positive exponents) or convergence (negative exponents) of nearby orbits along orthogonal directions in the state space. In numerical calculations of the LEs the asymptotic time averaging is usually accomplished by using a sufficiently long time to allow the averages of the exponents to converge within a set tolerance. Although less frequently used, probability densities (distributions) of the exponents, averaged over a much shorter time, also contain valuable dynamic information. Such distributions are made up of so-called finite-time, or local, Lyapunov exponents (LLEs)^4^,^5^,^6^.

For typical chaos the distribution of LLEs can be accurately fitted to a Gaussian function^4^,^5^, whereas for intermittent chaos, at crises, and for fully developed chaos, the distributions are characteristically non-Gaussian^7^. In the past the concept of LLEs has been used to characterise how secondary perturbations, localised in space, grow and spread throughout distributed dynamical (flow) systems with many degrees of freedom^8^. Variations on this technique, i.e. of co-moving or convective Lyapunov exponents^8^,^9^,^10^,^11^, continue to find new applications in a variety of different contexts, ranging from information theory^12^,^13^ to fluid flow (see, for example, ref. 14, and the references therein). The notion of finite-time Lyapunov exponents, averaged over initial conditions, has also been used to characterise transient chaos^15^.

In view of the fact that LLEs have been employed successfully to characterise many different types of nonlinear behaviour, it is natural to ask whether a dynamical system in a so-called chimera state^16^, may also possess a characteristic LLE distribution. Chimera states are a relatively new type of synchronisation phenomenon. They occur in systems of (usually) identical phase oscillators, which can be coupled, nonlocally^16^,^17^,^18^,^19^,^20^ (most frequently the case), globally (all-to-all)^21^ or even locally^22^. Depending on the nature of the coupling and the initial conditions, the oscillators may divide up into two or more spatially distinct groups, producing a spatiotemporal pattern which simultaneously contains domains of coherent and incoherent oscillations. However, such chimera states are fundamentally merely a different type of deterministic (hyper)chaos, having one or more positive LE(s)^23^,^24^.

Although the existence of chimera states was predicted more than a decade ago in the seminal paper by Kuramoto and Battogtokh^17^, experimental validation has only occurred recently^21^,^25^,^26^,^27^,^28^,^29^,^30^,^31^,^32^. Other than these fascinating experiments, chimera states may also be of physical importance in systems of Josephson junctions (JJs)^33^,^34^,^35^. Recently the spontaneous appearance of chimera states was found in numerical simulations of so-called SQUID metamaterials^36^. The superconducting quantum interference devices (SQUIDs) are made of JJs. A SQUID metamaterial is a one-dimensional linear array consisting of *N* identical SQUIDs, coupled together magnetically. The existence of a chimera state in this model suggests that they may soon be detected experimentally in existing one and two-dimensional SQUID metamaterials. At present there is a renewed and ongoing interest in these intriguing materials, which have even been proposed as a way of detecting quantum signatures of chimera states^37^.

Certain highly anisotropic cuprate superconductors, such as Bi_2_Sr_2_CaCu_2_O_8+*δ*_, contain natural arrays of intrinsic Josephson junctions (IJJs)^38^. At present there is a concerted effort being made towards achieving mutual synchronisation between stacks of IJJs, with the view of enhancing the power of the emitted radiation in the terahertz region^39^. In such systems the IJJs are coupled together in a way that is essentially nonlocal; a result of the breakdown of charge neutrality^40^, or a diffusion current^41^,^42^. IJJs could also provide a model for studying other synchronisation phenomenon, such as chaos synchronisation^43^,^44^. To this end, one of the difficulties that must first be overcome is related to the fact that, although the voltage across a stack of junctions can be measured with extreme precision, present experimental setups do not provide direct access to the voltages across individual junctions. Thus the states of the individual junctions have to be inferred, somehow, from indirect measurements. This is where the customary method of phase space reconstruction via embedding and the local function approximation^3^,^5^,^45^,^46^ may play an important role. In principle, the distribution of LLEs for a stack of intrinsic JJs could be obtained from a sufficiently long time series of the total voltage across the stack; thus, making it possible to detect the existence of a chimera state in the stack. At present there are several highly sophisticated techniques that could potentially be of use in this regard^47^,^48^,^49^,^50^.

While some previous studies of chimera states have computed their LEs^23^,^24^, to the best of our knowledge, only one study exists in which LLEs are used in connection with chimera states^51^. In ref. 51 the positions of the peaks in the distributions of LLEs were used to characterise the time evolution of intermittent chaotic chimeras occurring in coupled Kuramoto oscillators with inertia.

In view of the above considerations, LLE distributions may play an important role in characterising chimera states in general; but particularly in systems where the individual oscillators are not accessible experimentally. To explore this possibility we compute several distributions of LLEs corresponding to classic chimera states. We show that these distributions have a common characteristic shape which can be used to signal the occurrence of chimera states.

## Results

### Model equations

As a basis for our investigation we consider a general class of equations that support chimera states:


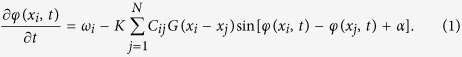


A similar form to Eq. (1) was originally derived by Kuramoto^52^ as an approximation to the complex Ginzburg-Landau equation, under weak coupling, when amplitude changes may be neglected. With relatively few exceptions, the form of Eq. (1) encompasses the majority of systems that have been considered in the literature on chimera states. (See Appendix A of the review article by Panaggio and Abrams^32^, and the references given therein.) It describes the dynamics of a non-locally coupled system of *N* phase oscillators, where *φ*(*x*_*i*_, *t*) is the phase of the *i*th oscillator, located at position *x*_*i*_. For identical oscillators the distribution of natural frequencies is given by^16^,^17^,^18^,^19^ *ω*_*i*_ = *ω*∀*i*. The function *G*(*x*) describes the non-local coupling between the oscillators^16^. *K* controls the overall coupling strength and the coefficients *C*_*ij*_ are either a coupling matrix, in the context of networks^53^, or else they are quadrature weights, in models where large numbers of oscillators have been considered^16^,^17^,^18^. In the latter models the oscillators are assumed to be continuously distributed throughout a one-dimensional spatial domain, leading to an integro-differential equation of the form


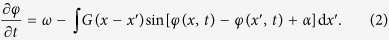


Since neither *C*_*ij*_ nor *G*(*x*_*i*_ − *x*_*j*_) depend on the phases, the Jacobian matrix of the system (1) can be expressed analytically as


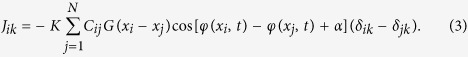


Equation (3) allows us to compute the LLEs via the standard algorithm, as explained in the first subsection of Methods.

### Distributions for intermittent chaos

**Figure 1:**
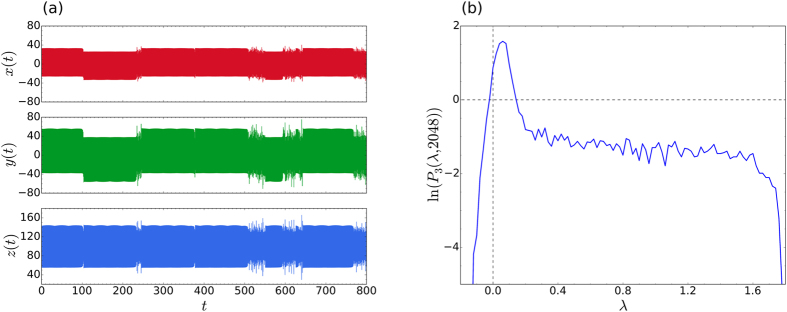
Characteristic distribution for intermittent chaos. (**a**) Time series showing intermittent chaos in the Lorenz system: 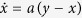, 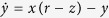, 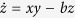, at parameter values *a* = 10, *r* = 100.796 and *b* = 8/3. In this simulation a time step Δ*t* = 1/64 and initial condition (1, 5, 10) were used. (**b**) Logarithmic plot of the corresponding probability density, *P*_3_(*λ*, 2048), for the distribution of (10000) maximal LLEs, *λ*, each obtained by averaging over 2048 time steps. The bin width was set to 0.02 and the total simulation time was 320000. An explanation of the notation *P*_3_(*λ*, 2048) is provided in the first subsection of Methods.

For the purposes of testing our numerical codes, and for making comparisons, several of the previously reported distributions^6^,^7^ were accurately reproduced. Since the characteristic distributions for intermittent chaos are closest to those found here for chimera states, we provide an example of intermittent dynamics in Fig. 1. This example was discussed in detail in ref. 7. As shown Fig. 1(a), the time series for the system shows that the system trajectory sporadically switches between almost periodic and chaotic behaviour. The trajectory in fact corresponds to classic (Type-I) tangent bifurcation intermittency^54^. In agreement with Fig. 6 of ref. 7 the characteristic distribution of LLEs consists of a superposition of two independent Gaussians, with stretched exponential interpolation between the two. Qualitatively, one can rationalise the shape of the distribution by considering that each Gaussian is roughly centred on the average value of the maximal LEs that would characterise each type of motion separately, i.e. if there was no switching. The exponential region, in-between the two Gaussian distributions, stems from samples that were taken over time intervals during which the system switched from one type of behaviour to the other.

### Characteristic distributions for chimera states

We now turn our attention to computing the LLEs for chimera states. For the purposes of comparison, we will make use of the four exemplary types of coupling functions discussed in the second subsection of Methods.

**Figure 2:**
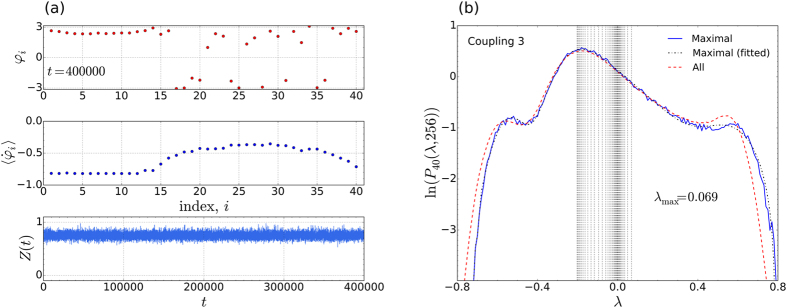
Characteristic distribution for a chimera state. (**a**) Instantaneous distribution of phases (top panel), the time averaged frequencies (middle panel), and a time series of the order parameter (bottom panel), for Coupling 3, as in ref. 23. Here 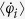 was averaged over 16384 time steps, with Δ*t* = 1/128. (**b**) Corresponding probability distribution of the maximal LLEs (blue, solid line) and all LLEs (red, dashed line), averaged over 256 time steps. The dotted (black) curve is the fitted distribution (see third subsection of Methods) for the maximal exponents, while the vertical dotted lines indicate the values of the (long-time averaged) LEs, the largest of these being *λ*_max_ = 0.069.

We begin by considering an interesting case of Eq. (1), that was analysed in detail by Wolfrum and Omel’chenko^23^. This case corresponds to Coupling 3, with *r* = 0.35, *C*_*ij*_ = 1/*N*, *N* = 40, *K* = 1, and *α* = 1.46. In Fig. 2(a) we show our results after 400000 simulation time units, for *α* = 1.478. Snapshots of the distribution in phases, *φ*_*i*_, the time averaged frequencies, 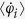, as well as the time series of the order parameter, 
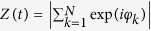
, clearly indicate that the oscillators are in a chimera state throughout the whole simulation. In Fig. 2(b) the corresponding distributions for the maximal LLE (blue, solid line) and all LLEs (red, dashed line), both averaged over 256 time steps, can be seen. As observed in previous calculations of the LLEs^7^, the distribution of all the exponents together has roughly the same shape as that of the maximal exponent alone. It is of course much smoother, due to the larger number of samples in the distribution, and is somewhat shifted towards the negative exponent side. As we will show, the general shape of the distribution in Fig. 2(b) appears to be a characteristic for *all* classic chimera states, arising from Eq. (1). The characteristic shape consists of an asymmetric Gaussian-like peak with a shoulder on either side.

At first the shape of the distributions for chimera states may seem quite similar to that of intermittent chaos [cf. Fig. 1(b)], but it is in fact distinctively different. For the case of intermittent chaos, the LLEs contributing to the relatively narrow peak, that can be seen on the left side of Fig. 1(b), come from samples that were taken during time intervals for which the trajectory stays entirely in the laminar phase^7^. The main peak for intermittency is therefore centred on a value of *λ* ≥ 0. By contrast, the much broader main peak of the characteristic distribution for a chimera state is always centred on some negative value of *λ*. Furthermore, as can be seen in Fig. 2(b), the chimera state distribution spans a more or less equal range in the positive and negative directions along the *λ* axis (measured from *λ* = 0).

To make the above qualitative observations more precise we have fitted a variety of distributions corresponding to chimera states (all obtained by solving Equation (1) at different parameters and for a selection of coupling functions), and found that all such distributions can be fitted accurately by a linear combination of four functions (see the third subsection of Methods). By contrast, distributions of LLEs for intermittent chaos can be fitted to a comparable accuracy by a linear combination of only two Gaussians and one exponential function. The distributions for chimera states do not lend themselves to a simple physical interpretation, as mentioned for the case of intermittent chaos. Therefore, a discussion of how the fluctuations of the dynamic instabilities are related to the peak and shoulders, will be postponed to the last subsection of Results.

In the next three subsections we show that the general shape of the characteristic distribution manifests itself over a wide range of parameters and different coupling kernels; as long as the system is in a stable chimera states, having only two domains, as in the classic cases^16^,^17^,^18^. When the dynamics of the system does not support such chimera states, the shape of the characteristic distributions become radically different, as we shall see below.

### Intermittent chimera states

Based on an analysis of the system in Fig. 2, at only one value (*α* = 1.46) of the phase-lag parameter^19^,^25^, Wolfrum and Omel’chenko^23^ concluded that, “Chimera states are chaotic transients”. Notwithstanding the experimental observations, there have also been several numerical counter-examples, in which stable chimera states could be simulated, independent of the population size or initial conditions^21^. Moreover, as Suda and Okuda^20^ have shown, the system in ref. 23 does support stable chimera states at other values of *α*. Therefore, in a general sense, the claim that chimera states are chaotic transients, is certainly not valid^20^.

**Figure 3:**
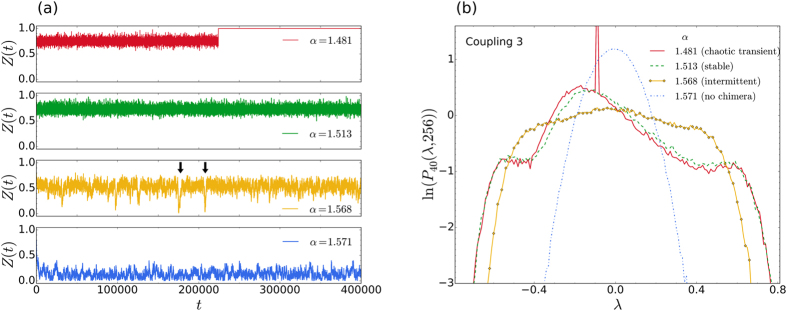
Transient and intermittent chimeras. (**a**) Time series of the order parameter 
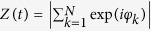
 for three different types of chimera states (upper three panels) and typical chaos (bottom panel). In the top panel (*α* = 1.481), the chimera state is transient and the oscillators all synchronise for *t* 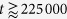. In the second panel from the top (*α* = 1.513), the state is stable, while in the third panel from the top (*α* = 1.568), it appears intermittently. The two downward pointing arrows, in the third panel from the top, show two time intervals during which the chimera state disappears. (**b**) The corresponding characteristic distributions of the maximal LLEs.

Consistent with the above findings, our numerical calculations also indicate that the system in Fig. 2 is capable of supporting different chimera states, depending on the value of *α*. In Fig. 3, we show our results for *α* = 1.481, 1.513, 1.568, and 1.571. In Fig. 3(a) the time series of the order parameters can be seen at the different values of *α*, while the corresponding distributions are shown in Fig. 3(b). Interestingly, in the time series for *α* = 1.568 (third panel from the top), at least time intervals (marked by the two black arrows) can be seen where the order parameter sporadically drops down to almost zero, i.e. intermittently the chimera state is lost. For the majority of the simulation time, however, *Z*(*t*) hovers around 0.6. Random spot checks on the phases and averaged frequencies show that the system is unambiguously in a chimera state for times when 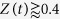. Thus, at *α* = 1.568, the chimera state is intermittent.

In Fig. 3(b) it can be seen that the characteristic distribution for the chimera state becomes flat-topped when the state is intermittent. Consistent with our expectations, once the chimera disappears completely, the distribution for the completely incoherent oscillations, is Gaussian in form – corresponding to typical chaos. On the logarithmic plot, in Fig. 3(b), it appears as a parabola. We emphasize that it is not out intention to study the transitions that occur here, as *α* changes. The purpose of the above considerations is merely to show that the distributions accurately reflect the different dynamic regimes that occur.

### Different averaging times

Although characteristic distributions of LLEs are stationary over a wide range of averaging times, different averaging times, used to compute the LLEs, can have an effect on the widths (and hence shapes) of the distributions^7^. Our numerical calculations are consistent with this expectation. For very short averaging times (comparable with one integration time step), the distributions are not stationary, i.e. they keep changing their shape, while in the asymptotic limit of (approximately) infinite averaging time, they become delta functions. However, provided these two extremes are avoided, the distributions are stationary and maintain their characteristic shapes over a relatively wide range of averaging times.

To investigate the effect of using different averaging times, we consider the classic chimera state that was reported by Abrams and Strogatz^16^. This case corresponds to Coupling 2, with *N* = 256, *A* = 0.995, and *α* = 1.39 in Eq. (2). As in ref. 16, we solve this system by using Simpson’s 3/8 quadrature rule^55^, for which *C*_*k*1_ = 3*h*/8, *C*_*k*2_ = 9*h*/8, *C*_*k*3_ = 9*h*/8, *C*_*k*4_ = 6*h*/8, *C*_*k*5_ = 9*h*/8, *C*_*k*6_ = 9*h*/8, *C*_*k*7_ = 6*h*/8, …, *C*_*kN*−1_ = 9*h*/8, *C*_*kN*_ = 3*h*/8. Here *h* = 1/(*N* − 1) is the separation between the oscillators.

**Figure 4:**
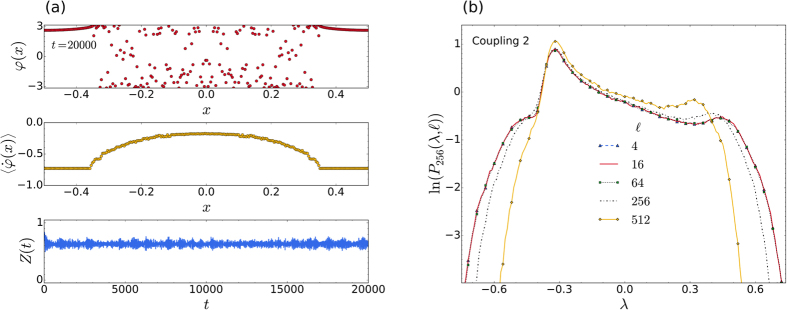
Different averaging times. (**a**) The chimera state for the system described in ref. 16, showing a snapshot of the phases (top panel) and the averaged frequencies (middle panel), after an integration time of 20000 dimensionless units. The averaging was done over 16384 time steps, each of duration Δ*t* = 1/128. The bottom panel shows the time series of the order parameter *Z*(*t*). (**b**) Comparison of the characteristic distributions of all LLEs for the system in (**a**), obtained by averaging the LLEs over 4, 16, 64, 256, and 512 time steps, respectively. The distributions for the lower three averaging times practically coincide.

Figure 4(a) shows the distribution of phases, averaged frequencies, and the time series of the order parameter for this chimera state. More pertinent to the current discussion, Fig. 4(b) shows the distributions of the LLEs, obtained by averaging over successively larger time intervals, starting from an averaging time of 4/128 = 0.03125, which corresponds to only 4 time steps. All the distributions shown are stationary (in time), and their characteristic shape remains clearly discernible for a range of averaging times, spanning about two orders of magnitude. We notice here that the left shoulder of the characteristic distribution is somewhat less prominent (even at the shorter sampling times), in comparison to the previously seen characteristic distributions. This is simply because the position of the main asymmetric peak, is now much closer to the left shoulder. It will also be noticed that the main peak is more asymmetric and sharper than before, as it now overlaps the left shoulder. As we will show in the next subsection, the position of the main peak, relative to the two shoulders, depends in a regular way on the value of the phase-lag parameter, within the approximate range: 1.3 < *α* < 1.57.

From our simulations we have deduced a practical guideline for estimating the upper bounds of averaging times that still produce useful (easily recognisable) characteristic distributions. It appears that, as long as the averaging time is substantially less than the average rotational period of the coherent bunch of oscillators in the system, the characteristic shape of the distribution is easily discernible. In Fig. 4(a), for example, the average rotational period of the coherent bunch can be seen to be approximately 2*π*/0.72 ≈ 8.7, while the largest averaging time shown in Fig. 4(b) (512/128 = 4) is roughly half this value.

### Distributions for different *N* and *α*

**Figure 5:**
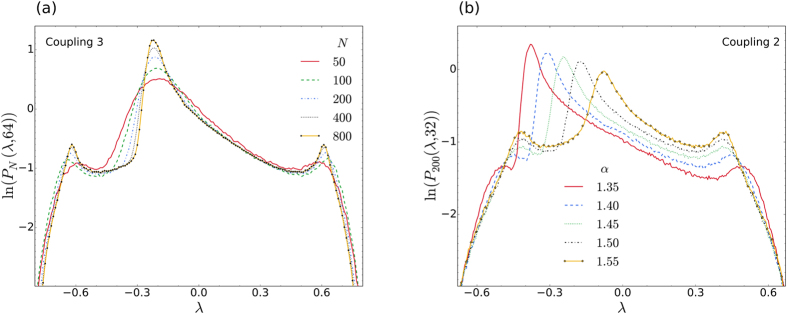
Variation with system size and phase-lag parameter. (**a**) Comparison of the distributions for chimera states as the number of oscillators, *N*, is successively doubled. Other than the change in *N*, all the parameters for this system are the same as for Fig. 2. The same system, for *N* = 200, *K* = 2 and *α* = 1.46, was studied in ref. 62, where the position of the incoherent bunch of oscillators was shown to execute Brownian motion; a finite-size effect that vanishes in the thermodynamic limit^62^. Intriguingly, here the successive characteristic distributions appear to be converging for large *N*. (**b**) Distributions at different *α*, for Coupling 2 with *C*_*ij*_ = 1/*N*, *A* = 0.995 and *K* = 1. As *α* increases to its maximum (*π*/2 ≈ 1.57), the position of the main peak systematically shifts, from close to the left shoulder, towards the middle of the two shoulders.

We now consider how the LLE distributions vary in response to changes in the system size and phase-lag parameter. Figure 5(a) shows the distributions obtained for the system of Fig. 2 (with *α* = 1.46), as the number of oscillators is successively doubled, starting from *N* = 50. In all four cases we observe that the distributions maintain their characteristic shape, as described previously. However, as *N* increases, the two shoulders and central peak, become more pronounced, and the overall distribution appears to converge towards the (computationally unobtainable) limiting case of *N* → ∞, i.e. we see that the overall change in successive distributions become less apparent, even though *N* is doubled each time.

In Fig. 5(b) we consider again Coupling 2, but this time at six different phase-lag parameters. Here one can clearly see how the position of the main asymmetric peak shifts in response changes in *α*. At the lowest value the main peak is positioned close to the left shoulder, while at the high value, it is almost midway between the two shoulders. It should be noted that the main peak never moves beyond these two extremes, because the chimera state becomes unstable for slightly smaller or larger values.

### Other coupling schemes

**Figure 6:**
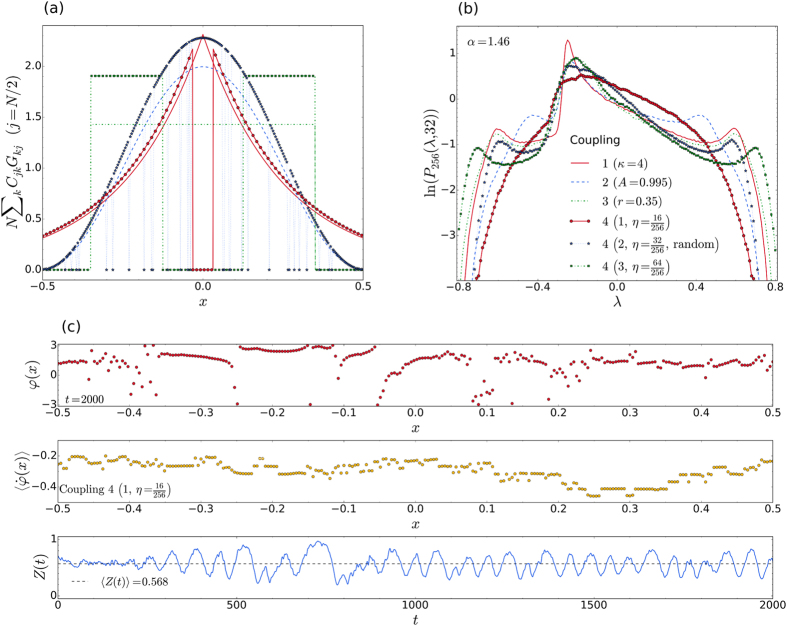
Other types of coupling. (**a**) Illustration of the six different couplings corresponding to the adjacent distributions. (**b**) LLE distributions corresponding to the couplings in (**a**). All the distributions, except the exponential coupling 4(1), which has a fraction, *η* = 16/256, of nearest links removed, are of the characteristic shape. Note that (**a**,**b**) share the same legend. (**c**) Snapshot of the phases (top panel), averaged frequencies (middle panel), and the time series of the order parameter (bottom panel) for the distribution 4(1), which does not conform to the characteristic shape for a chimera state. Here we see that the nonconforming case corresponds to a multiple, breathing chimera, i.e. one consisting of more than two different domains, showing significant oscillations in *Z*(*t*).

At present, chimera states have been observed in simulations of Eq. (1), for a variety of different couplings. Different studies have also revealed several new types of chimeras, including breathing^56^, virtual^28^, intermittent chaotic^31^,^51^, travelling^57^, and even imperfect chimeras^30^. Clearly it is beyond the scope of the present work to investigate the possibility of using the characteristic distributions to identify all these other types of chimeras. In this subsection we show that the different couplings all lead to the characteristic shape, provided that the chimeras are of the traditional type, i.e. they consist of only two domains and have a relatively constant (in time) order parameter. Figure 6 shows a comparison of the distributions for six different coupling schemes, including one that produces a multiple breathing chimera (see Fig. 6(c)). As in the case of the intermittently appearing chimera, this multiple breathing chimera has an uncharacteristic shape.

### Dynamic origin of the characteristic shape

So far we have not considered how the observed characteristic shape of the distributions for chimera states comes about. In particular, one would like to understand the origin of the asymmetric central peak, with its two shoulders. How, precisely, may the peaks in the characteristic distribution be related (if at all) to the constituent domains of the chimera, i.e. the coherent domain, the incoherent domain, and the dividing transitional domain? One way to address this question formally, could be via the theory of covariant Lyapunov vectors (CLVs)^58^,^59^,^60^,^61^. By this relatively new technique, the Lyapunov exponents may be numerically calculated in the directions of a spanning set of covariant vectors, which naturally co-rotate with the flow; thus, making it possible to keep track of how the different degrees of freedom may be contributing to the distribution. However, the efficient implementation of one or more of the available algorithms for this purpose would, in and of itself, constitute a whole new research project.

**Figure 7:**
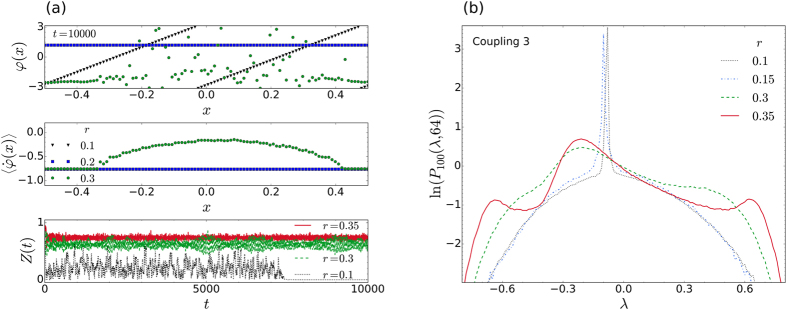
Dynamic origin of the characteristic shape. (**a**) Distribution of the phases (top panel), average frequencies (middle panel) and the time series of the order parameters (bottom panel), for the same system as in Fig. 5(a), with *N* = 100 and at different coupling ranges *r*. For 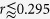 there is no persistent chimera state, and the system eventually evolves to a splay or synchronous state, as shown for *r* = 0.1 and 0.2, respectively. For 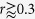 chimera states appear, as shown for *r* = 0.3 and 0.35. As the coupling range increases from 0.3 to 0.35 we observe that the amplitudes of the fluctuations in the time series of the corresponding order parameters for the chimera states decrease, and the average values of the order parameters increase. (**b**) The distributions of LLEs corresponding to four different coupling ranges. The sharp Gaussian peaks that can be seen in the distributions for *r* = 0.1 and 0.15 are due to contributions from the end portions of the (synchronous or splay state) trajectories. As the coupling range exceeds the threshold for the formation of chimeras (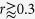) the corresponding distributions assume the characteristic shape.

Here, to develop a more intuitive (phenomenological) understanding of how the shape in the characteristic distribution comes about, we have performed many additional simulations for the same system that was discussed in connection with Fig. 5(a). Specifically, a total of 251 simulations were performed at the coupling ranges *r* = 0.1, 0.101, 0, 102, …, 0.349, 0.35, all at *N* = 100. These results are summarized by Fig. 7. At the two smallest coupling ranges shown (*r* = 0.1 and 0.15), no chimera state could be simulated, irrespective of the optimally prepared initial conditions that were employed. Instead, as summarised in Fig. 7(a), for the case *r* = 0.1, we found that the system rapidly evolves to a state where all the oscillators are oscillating incoherently. This state persists for only a finite time (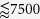), as can be seen in the lower panel of Fig. 7(a), before changing either to a synchronous state, or a splay state, for which there is a constant phase separation between all the oscillators. The corresponding distributions of LLEs, which are shown in Fig. 7(b), provide a good indication that the same basic dynamics giving rise to the synchronous or splay state may also be contributing to the central peak in the characteristic distribution. In support of this assertion, in Fig. 7(b), one sees that the distributions corresponding to *r* = 0.1 and 0.15 consist of two Gaussians; the broader Gaussian originates from LLEs sampled during the incoherent part of the trajectory, while the much narrower peak (spike) originates from the end part. Furthermore, as the coupling range increases, the spikes systematically shift towards the position where the asymmetric central peak eventually manifests itself for the larger coupling ranges (as shown for *r* = 0.3 and 0.35), for which chimeras do occur. Thus there are indications that the central peaks seen in the characteristic distributions may be related to degrees of freedom corresponding to the coherent domain. At the same time, it should be remembered that, for a chimera state, there is an interdependence between the two (mutually existing) domains: the coherence in the one domain is supported by the incoherence in the other, and vice versa.

Two shoulders on either side of the asymmetric peak may also reflect the delicate balance on which the chimera’s existence relies. Really, due to the form of the coupling in this model the directions of growth and contraction of vectors in the tangent space are governed by a Jacobian matrix which is essentially of the same form as the coupling itself. Each element of the Jacobian matrix [see Eq. (3)] is merely phase shifted by *π*/2. Furthermore, the maximum and minimum values that elements in the Jacobian matrix can assume are limited by the two extrema of the cosine function. This could explain why there are two shoulders in the characteristic distribution; one corresponding to pairs of oscillators at or near the maximal attraction, the other to pairs which are near maximal repulsion.

## Discussion

We have calculated the probability distributions of local Lyapunov exponents (LLEs) corresponding to several classic chimera states. We found that these distributions all had a specific shape, which can be described as consisting of a main asymmetric Gaussian-like peak, with a shoulder to either side. Generally the position of the main peak is located nearer to the left shoulder.

In principle, knowledge of the expected shape of the characteristic distribution could be used to identify the occurrence of chimera states in real physical systems, particularly in those for which it may not be possible to measure, directly the phases of all the oscillators. In such cases, we envisage that advanced embedding techniques could be employed to extract the characteristic distribution of LLEs, which would then be a useful signature of the chimera state (for it would otherwise have been undetectable). The present results may thus find application in a variety of systems where chimera states are thought possible, but are not directly/easily observable. A comprehensive review of the relevant real-world systems may be found in ref. 32 and the references therein.

Although we have provided a qualitative discussion of how the shape of the characteristic distribution may be related to the nature of the coupling and the different domains of oscillation in the chimera state, the problem of obtaining a more quantitative (mathematical) proof for this question remains open. It has been suggested that the method of covariant Lyapunov vectors^58^,^59^,^60^,^61^, which is increasingly being applied to a wide range of nonlinear models, may provide a suitable framework for such future investigations.

The case of intermittently appearing chimeras, as discussed in connection with Fig. 3, is currently of particular interest, in view of the fact that intermittent chaotic chimeras have only recently been reported in the literature for a much more complicated system^31^. This system consisted of two symmetrically coupled populations of *N* oscillators, where one population is synchronised and the other jumps erratically between laminar and turbulent phases^31^. The present considerations indicate that such intermittency also arises in the original prototype system for chimera states, although it was not previously reported.

## Methods

### Calculation of the LLE probability density

Consider an autonomous *N*-dimensional continuous dynamical system of the form 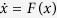. The probability density (distribution) of the 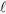
th LLE, 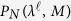, is defined so that 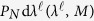 equals the probability that 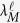 takes on a value between 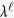 and 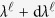, where


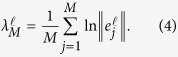


Here 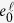 is the 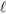
th vector of the initial set of orthonormal vectors, and 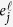 is its time evolved value under the action of the linearised equations of motion after *j* time steps. Thus the 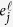 are obtained by solving the equations


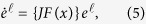


where *J* is the system Jacobian. Notice that 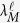 is the 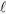
th exponent averaged over *M* time steps. Because the vectors 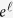 diverge exponentially in magnitude, and tend to align themselves along the local direction of most rapid growth, their exact directions may rapidly become numerically indistinguishable. To overcome this difficulty, as explained in refs 1, 2, 3, Gram-Schmidt orthogonalisation can be performed on the set of frame vectors. In the present work we have performed the Gram-Schmidt orthogonalisation after every time step.

As a starting point for our calculations we made use of the Fortran implementation provided by Wolf, Swift and Swinney^3^, for the case when the system Jacobian matrix is known analytically. Their code required relatively few modifications. Other than changing the system equations, the code was rewritten in such a way that it could be called repeatedly over successive segments of the trajectory, at the same time returning a time series of the trajectory, from which the averages 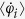 could be computed. To integrate the system, i.e. Eq. (1) and its linearisation, we employed a fifth-order Runge-Kutta integration scheme, with a fixed time step, Δ*t* = 1/64, unless indicated differently. To check the numerical convergence of the calculations, all the calculations were repeated with the time step halved.

### Coupling Functions

Arguably, a widely studied model of chimera states consists of *N* identical (*ω* = 0) nonlocally coupled phase oscillators which are distributed uniformly over an interval, with periodic boundary conditions. Within this model, different normalisations of the coupling function *G*(*x*), and the choice of the interval length itself, can lead to different coupling ranges and strengths. In the literature on chimera states, several different interval lengths and normalisations have been chosen. This makes it difficult to obtain a clear understanding of how systematic changes in the system parameters, and different forms of the coupling, may lead to the wide variety of dynamical behaviours that have so far been reported.

In the present work, we fix the interval to be 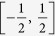 and consistently make use of the normalisation 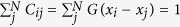 (*i* = 1, 2, …, *N*). For convenience, more details about our exemplary coupling functions are provided below. We list these functions in chronological order, as they first appeared in the literature.

#### Coupling 1: exponential

This exponentially decaying form of the coupling function was derived in the earliest report on chimera states, by Kuramoto and Battogtokh^17^, where 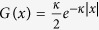 (with *κ* = 4) was normalised to unity over the entire real line, even though the oscillators were distributed only over the interval [0, 1]. When normalised to unity over 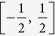, we find that


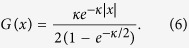


#### Coupling 2: co-sinusoidal

This co-sinusoidal form of coupling was devised, in the seminal paper by Abrams and Strogatz^16^, as a form which allowed Eq. (2) to be solved analytically. In the work by Abrams and Strogatz^16^,^18^, *G*(*x*) is normalised to unity over the interval [−*π*, *π*]. When normalised over the interval 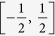,


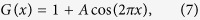


where 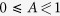, as usual.

#### Coupling 3: rectangular

The rectangular coupling function was introduced in the literature on chimera states by Omel’chenko *et al.*^62^, where the interval [−1, 1] was considered. It is given by


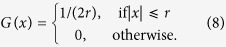


Clearly this function is normalised over any interval that is equal to or wider than twice the coupling length *r*, but one of course has to adjust *r* according to the chosen interval length. For example, the coupling range of *r* = 0.7, that was used in ref. 62, is equivalent to *r* = 0.35, over the interval 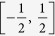.

We note that the model equation that was used in ref. 62 corresponds to *K* = 2 and *C*_*ij*_ = 1/*N* in Eq. (1). It may therefore be viewed as the result of approximating the integral in Eq. (2) by a left Riemann sum, with an added factor of 2 in the coupling strength *K*.

#### Coupling 4: systematic or random link removal

In networks the idea of systematically or randomly removing the coupling (or links) between the oscillators (nodes) may also be related to chimera states. Recently, for example, it has been shown that such perturbations of the coupling configuration can lead to multicluster chimera states^53^,^63^. In the article by Nan Yao *et al.*^63^, the emergence of multicluster chimera states was reported to be a consequence of the systematic removal of a certain fraction (*η* = 2*L*/*N*) of nearest links in the network. In the present work, as in ref. 63, the coupling matrix *C*_*ij*_ (in Equation (1)) is used to control the number of links between the oscillators. In particular, to remove the nearest *L* links from the oscillator *i*, we set


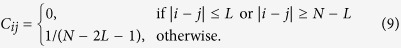


We use a similar scheme to remove a certain fraction of links, randomly, always ensuring that the normalisation of *C*_*ij*_ is maintained. In passing, we note that, in ref. 63, the coupling matrix is not consistently normalised to unity. There, the 
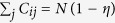
, leading to a decrease in the coupling strength (equivalent to setting *K* = 2*π*(1 − *η*) in the present work), as *L* increases (*L* = 1, 2, …, *N*/2).

### Fitting the characteristic distribution

It is found that the characteristic distribution of LLEs for chimera states can be fitted accurately by a linear combination of the form *g*_1_(*x*) + *f*(*x*) + *g*_2_(*x*) + *B* exp(−*x*/*τ*), where *g*_1_ and *g*_2_ are Gaussians given by


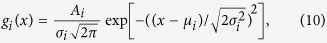


and *f*(*x*) is an exponentially modified Gaussian, given by


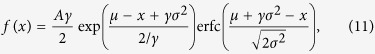


where erfc is the complementary error function^55^. To perform the fitting we made use of the Python package lmfit^64^, which stands for Non-Linear Least-Squares Minimisation and Curve-Fitting for Python. The initial parameters were chosen so that the Guassians were roughly centred on the two shoulders of the distribution, with *f*(*x*) on the central main peak.

As an example, the fitted distribution has been plotted in Fig. 2(b) as a dotted (black) line. Although the fitted distributions have not been shown in subsequent figures, fitting curves of comparable accuracy (reduced *χ*^2^ < 0.002) have been obtained with the same formula for all the chimera state distributions discussed in this article.

## Additional Information

**How to cite this article**: Botha, A. E. Characteristic distribution of finite-time Lyapunov exponents for chimera states. *Sci. Rep.* **6**, 29213; doi: 10.1038/srep29213 (2016).
